# Serum Uric Acid Levels are Associated with Cardiometabolic Risk Factors in Healthy Young and Middle-Aged Adults

**DOI:** 10.5935/abc.20180197

**Published:** 2018-12

**Authors:** Thaís da Silva Ferreira, Julia Freitas Rodrigues Fernandes, Luciene da Silva Araújo, Lívia de Paula Nogueira, Priscila Mansur Leal, Vanessa Parada Antunes, Maria de Lourdes Guimarães Rodrigues, Debora Cristina Torres Valença, Sergio Emanuel Kaiser, Márcia Regina Simas Torres Klein

**Affiliations:** 1 Universidade Federal do Estado do Rio de Janeiro (UNIRIO), Rio de Janeiro, RJ - Brazil; 2 Universidade do Estado do Rio de Janeiro (UERJ), Rio de Janeiro, RJ - Brazil

**Keywords:** Uric Acid/metabolism, Oxidative Stress, Inflammation, Endothelium/ dysfunction, Adults

## Abstract

**Background:**

Observational studies have highlighted an association between serum uric acid (SUA) levels and cardiovascular risk factors. Despite the growing body of evidences, several studies were conducted in older individuals or in carriers of diseases susceptible to affect SUA levels and cardiometabolic risk markers.

**Objective:**

To evaluate the relationship of SUA with body adiposity, metabolic profile, oxidative stress, inflammatory biomarkers, blood pressure and endothelial function in healthy young and middle-aged adults.

**Methods:**

149 Brazilian adults aged 20-55 years, both sexes, underwent evaluation of body adiposity, SUA, fasting glucose and insulin, lipid profile, malondialdehyde (MDA), high sensitivity C-reactive protein (hs-CRP), adiponectin, blood pressure and endothelial function. Endothelial function was assessed by the reactive hyperemia index (RHI) derived from peripheral arterial tonometry method. Participants were allocated in two groups according to SUA levels: control group (CG; n = 130; men ≤ 7 mg/dL, women ≤ 6 mg/dL) and hyperuricemia group (HG; n = 19; men > 7 mg/dL, women > 6 mg/dL). A P-value < 0.05 was considered statistically significant.

**Results:**

After adjustment for confounders, participants in HG compared with those in CG displayed higher body mass index (BMI): 34.15(33.36-37.19) vs.31.80 (26.26-34.42) kg/m^2^,p = 0.008, higher MDA: 4.67(4.03-5.30) vs. 3.53(3.10-4.07) ng/mL, p < 0.0001 and lower RHI: 1.68 ± 0.30 vs. 2.05 ± 0.46, p = 0.03). In correlation analysis adjusted for confounders, SUA was positively associated (p < 0.05) with BMI, waist circumference, LDL-cholesterol, triglycerides and MDA, and negatively associated (p < 0.05) with HDL-cholesterol, adiponectin and RHI.

**Conclusions:**

This study suggests that in healthy young and middle-aged adults higher SUA levels are associated with higher body adiposity, unfavorable lipid and inflammatory phenotype, higher oxidative stress and impaired endothelial function.

## Introduction

Cardiovascular diseases (CVD) are the leading causes of death in the world. According to World Health Organization, ischemic heart disease and stroke together accounted for 15 million deaths in 2015.^1^ Therefore, it is important to identify early and cost-effective markers of CVD risk.

Uric acid is the final product of endogenous and dietary purine metabolism.^2^ In several cross-sectional and longitudinal observational studies, elevated serum uric acid (SUA) levels have been associated with increased risk for cardiovascular events and mortality, as well as with cardiovascular risk factors such as hypertension, obesity, metabolic syndrome, insulin resistance and dyslipidemia.^3^ Increased SUA concentration has also been positively correlated with surrogate markers of CVD: impaired endothelial function, increased carotid intima-media thickness and aortic stiffness.^4-11^

It is noteworthy that the majority of the studies aimed at evaluating the relationship of SUA with vascular function and/or cardiometabolic markers were conducted in postmenopausal women, older individuals and/or in individuals with renal impairment or CVD risk factors (ex. hypertension and diabetes).^3,5-11^ Therefore, the participants included in many previous studies were more likely affected by a compromised cardiocirculatory and/or metabolic status which would represent a confounding factor in the association between SUA and cardiometabolic risk factors.

The purpose of this study was to evaluate the relationship of SUA with body adiposity, metabolic profile, inflammatory biomarkers, oxidative stress, blood pressure and endothelial function in a sample of healthy young and middle-aged adults.

## Methods

The present cross-sectional study was conducted at the Discipline of Clinical and Experimental Pathophysiology (CLINEX), located at Pedro Ernesto University Hospital, Rio de Janeiro State University.

Potential participants were recruited in the waiting room of the Departments of orthopedics, plastic surgery and gynecology. Inclusion criterion was age between 18-55 years.

Exclusion criteria were smoking; use of dietary supplements; use of medications susceptible to interfere in body weight, metabolic profile and blood pressure; use of α-adrenergic blocking agents; recent changes (within previous 6 months) in body weight (> 3 kg), in dietary intake and in intensity or frequency of physical exercise; diagnosis of diabetes mellitus, hypertension, dyslipidemia (with drug treatment) and kidney disease; clinical history of thyroid dysfunction, angina pectoris, peripheral vascular disease, peripheral neuropathy, heart failure, liver failure, chronic pulmonary disease, myocardial infarction and stroke; and finger deformity that would prevent the proper use of the sensors necessary to evaluate endothelial function. Pregnant or lactating women were not allowed into the study.

Subjects who met eligibility criteria and agreed to take part in the study were scheduled to arrive at the CLINEX Laboratory between 08:00 and 10:00h a.m. after a 12h fasting period and abstinence from alcohol for 3 days. While fasting, they were submitted to clinical, nutritional, laboratory and endothelial function evaluations.

### Nutritional assessment

A semi-quantitative food frequency questionnaire (FFQ) was used to assess the usual dietary intake of energy, proteins, carbohydrates, lipids, cholesterol, fiber and calcium over the previous 6 months. This FFQ containing 80 items and usual portions was developed for the Brazilian population based on commonly consumed foods.^12^ Alcohol consumption was considered when reported frequency equaled one or more time per week.

Height was measured by a stadiometer accurate to ± 0.5 cm and weight was obtained with a calibrated scale accurate to ± 0.1 kg (Filizola S.A., São Paulo, SP, Brazil) after participants without shoes and wearing light clothing, attempted to empty their bladder. Body mass index (BMI) was calculated using the standard equation (kg/m^2^). Waist circumference (WC) was measured in the standing position midway between the lower margin of the last rib and the iliac crest at mid-exhalation. Hip circumference was measured at the widest point of the hip/buttocks area with the measuring tape parallel to the floor. Waist-to-hip ratio was determined by dividing WC (cm) by hip circumference (cm). Waist-to-height ratio was obtained by dividing WC (cm) by height (cm). The anthropometric measurements were taken twice and mean values were used in all analysis.

### Laboratory parameters

Aliquots of plasma and serum were stored at -80ºC as appropriate for laboratory determinations. Laboratory parameters included fasting circulating levels of uric acid, glucose, insulin, urea, creatinine, lipid profile, high-sensitivity C reactive protein (hs-CRP), adiponectin and malondialdehyde (MDA).

Serum concentration of uric acid was determined by enzymatic colorimetric method and urea and creatinine by kinetic method. Fasting plasma glucose was measured by hexokinase method. Fasting plasma insulin levels were determined by the enzyme-linked immunosorbent *assay* (ELISA) method using the commercially available specific kit (EMD Millipore Corporation Billerica, MA, USA). Insulin resistance status was assessed by homeostasis model assessment of insulin resistance (HOMA-IR) index, calculated as fasting insulin (µU/mL) × fasting plasma glucose (mmol/L)/22.5.^13^

Total cholesterol and triglycerides (TG) were assessed by enzymatic method (cholesterol oxidase-peroxidase and glycerol phosphate oxidase-peroxidase, respectively). High density lipoprotein (HDL)-cholesterol was determined by a direct method. Low density lipoprotein (LDL)-cholesterol was estimated by Friedewald’s formula.^14^

Circulating levels of hs-CRP and adiponectin were chosen as markers of inflammatory state and their serum concentration determined respectively by turbidimetry (BioSystems, Barcelona, Spain) and ELISA (EMD Millipore Corporation Billerica, MA, USA). Serum levels of MDA, regarded as a *measure* of oxidative stress, were determined by ELISA method using a commercial kit (USCN Life Science Inc., Missouri, USA).

### Blood pressure and heart rate

Blood pressure and heart rate were recorded after a resting period of 10 minutes by a calibrated automatic sphygmomanometer: OMRON^®^ Model HEM-742INT (Omron Healthcare, Lake Forest, IL, USA).The first reading was discarded and the mean of 3 consecutive measurements, taken with a 3 - minute interval in the non-dominant arm, was used in the study. An appropriate arm cuff was used and the patient was instructed to stay seated, legs uncrossed, feet on the floor, leaning back in his chair with the arm at heart level, free from tight clothing, supported with the palm facing up and elbow slightly flexed.

### Endothelial function

Endothelial function was evaluated by peripheral artery tonometry (PAT) method, using Endo-PAT 2000^®^, a finger plethysmographic device (Itamar Medical, Caesarea, Israel). This is a non-invasive method that offers the possibility of an easy and rapid assessment of vascular function in which data are analyzed independently of the examiner. Alterations in pulsatile arterial volume detected by PAT have shown good correlation with flow-mediated dilatation measurement.^15^

The measurements were performed through fingertip probes placed on both index fingers. A 5 min measurement was taken at baseline. Sequentially, arterial flow was occluded by a cuff applied to the non-dominant arm, and inflated to 60 mmHg above systolic blood pressure, but never below 200 mmHg. The cuff was rapidly deflated after a 5-min occlusion period, to allow reactive hyperemia. The following 5 min were also recorded. The other arm served as a control and the difference between the two arms was used by Endo-PAT 2000^®^ software to automatically calculate the reactive hyperemia index (RHI).

### Statistical methods

Participants were stratified into two groups according to their SUA levels: control group and hyperuricemia group. The control group was formed by men and women presenting SUA ≤ 7 and ≤ 6 mg/dL, respectively, whilst the hyperuricemia group consisted of men and women with SUA > 7 and > 6 mg/dL, respectively.

Mean values and standard deviations were used to summarize continuous variables with normal distribution, while median and interquartile interval were used to summarize variables with non-normal distribution. Normality was tested by the Shapiro-Wilk test. The differences between groups were analyzed using unpaired Student’s t-test or Mann-Whitney test, as appropriate. Multiple regression was used to adjust for confounding factors, including age, gender and BMI. Categorical variables were expressed as percentage and compared by X^2^ test.^2^

Pearson’s or Spearman’s correlation coefficient was performed to analyze the degree of association of SUA and anthropometric indices, laboratory variables, blood pressure and endothelial function among all participants. Partial correlations controlled for different confounders, including parameters of body adiposity, were also used.

Statistical analyses were carried out through STATA version 12.0 (STATA Corp., College Station, TX, USA) and a P-value < 0.05 was considered statistically significant. Sample size was determined by convenience.

## Results

A total of 149 volunteers were included in the statistical analysis. Their average age was 35.02 ± 9.57 years, mean BMI of 31.17 ± 5.87 kg/m^2^and mean SUA levels were 4.67 ± 1.41 mg/dL. Participants in control group (n = 130) and in hyperuricemia group (n = 19) were comparable in age, gender, alcohol intake, physical activity, ethnicity and serum levels of urea and creatinine (Table1).

**Table 1:** Comparison of participants’ characteristics according to diagnosis of hyperuricemia

	Control group (n = 130)	Hyperuricemia group (n = 19)	p
Male sex, n (%)	19 (14%)	6 (32%)	0.06
Alcohol intake, n (%)	44 (34%)	9 (47%)	0.30
Physical activity, n (%)	19 (14%)	2 (13%)	0.93
Non-white ethinicity, n (%)	83 (64%)	14 (74%)	0.40
Age (years)	34.00 (27.00 - 42.50)	31.00 (27.00 – 43.00)	0.93
Serum uric acid (mg/dL)	4.32 ± 1.09	7.18 ± 0.67	< 0.001
Serum urea (mg/dL)	29.31 ± 17.02	29.73 ± 8.28	0.84
Serum creatinine (mg/dL)	0.80 ± 0.17	0.83 ± 0.16	0.56

Values as mean ± standard deviation for normal distribution or as median (interquartile interval) for not normal distribution or absolute values (%). p: Control group vs.Hyperuricemia group.

Dietary intake of energy and carbohydrates were significantly higher in hyperuricemia group than in control group, while the intake of monounsaturated fatty acids was significantly lower. However, after adjustments for age, sex and BMI these differences were no longer significant (Table 2).

**Table 2:** Comparison of participants’ usual dietary intake according to diagnosis of hyperuricemia

	Control group (n = 130)	Hyperuricemia group (n = 19)	p	p*
Energy (kcal/day)	1647.5 (1250.3 – 2099.0)	2212.2 (1543.4 – 2934.4)	0.02	0.77
Protein (g/day)	75.7 (63.5 – 93 9)	77.6 (67.1 – 112.8)	0.73	0.80
Carbohydrates (g/day)	196.0 (143.2 – 266.9)	296.2 (202.5 – 412.0)	0.01	0.49
Lipids (g/day)	60.3 (44.0 – 78.9)	75.9 (47.9 – 106.1)	0.23	0.71
Saturated fatty acids (g/day)	24.4 (18.4 – 31.0)	25.6 (14.9 – 29.5)	0.84	0.12
Poliunsaturated fatty acids (g/day)	7.1 (5.4 – 9.6)	8.8 (6.4 – 12.2)	0.15	0.75
Monounsaturated fatty acids (g/day)	11.1 (7.5 – 15.7)	7.0 (4.2 – 9.9)	0.01	0.12
Cholesterol (mg/day)	286.7 (207.1 – 425.0)	231.6 (152.8 – 417.7)	0.18	0.12
Fiber (g/day)	19.3 (14.9 – 25.4)	18.3 (12.7 – 19.6)	0.54	0.78
Calcium (mg/day)	706.6 (541.0 – 959.5)	773.1 (642.8 – 952.5)	0.46	0.55

Values as median (interquartile interval). p: Control group vs.Hyperuricemia group.

* p: Control group vs.Hyperuricemia group, after adjustment for age, sex, and body mass index.

Individuals in hyperuricemia group compared with those in control group exhibited significantly higher BMI even after controlling for age and sex, regarded as variables able to interfere with theses parameters (Table 3). WC was higher in hyperuricemia group after controlling for age but not after further adjustment for sex.

**Table 3:** Comparison of participants’ anthropometric parameters according to diagnosis of hyperuricemia

	Control group (n = 130)	Hyperuricemia group (n = 19)	p	p*	p**
Body mass index (kg/m^2^)	31.80 (26.26 – 34.42)	34.15 (33.36 – 37.19)	0.006	0.003	0.008
Men	32.30 (30.60 – 34.61)	36.53 (33.50 – 37.19)	0.03	0.04	-
Women	31.68 (24.17 – 34.10)	33.90 (33.36 – 36.13)	0.04	0.05	-
Waist circumference (cm)	98.75 (85.60 – 106.00)	105.60 (99.00 – 112.00)	0.05	0.03	0.12
Men	106.00 (102.50 – 114.50)	112.25 (106.00 – 113.00)	0.19	0.18	-
Women	97.00 (82.50 – 103.50)	99.50 (96.50 – 106.00)	0.26	0.38	-
Waist-to-hip ratio	0.89 (0.81 – 0.94)	0.89 (0.82 – 0.93)	0.76	0.70	0.69
Men	0.95 (0.93 – 0.96)	0.92 (0.89 – 0.95)	0.10	0.27	-
Women	0.86 (0.79 – 0.92)	0.87 (0.82 – 0.93)	0.96	0.68	-
Waist-to-height ratio	0.61 (0.55 – 0.65)	0.63 (0.59 – 0.66)	0.12	0.08	0.13
Men	0.62 (0.59 – 0.65)	0.64 (0.61 – 0.66)	0.46	0.36	-
Women	0.60 (0.52 – 0.63)	0.63 (0.59 – 0.66)	0.22	0.32	-

Values as median (interquartile interval). p: Control group vs.Hyperuricemia group.

* p: Control group vs.Hyperuricemia group, after adjustment for age.

** p: Control group vs.Hyperuricemia group, after adjustment for age and sex.

Comparative analysis of biochemical variables between hyperuricemia group and control group showed similar serum levels of glucose, insulin, HOMA-IR, total cholesterol, LDL-cholesterol, TG and hs-CRP. HDL-cholesterol was higher in control group only before adjustments for age, sex and BMI (Table 4). As compared to subjects in control group, those in hyperuricemia group still exhibited significantly lower levels of MDA, after adjustments for potential confounders (age, sex and BMI) (Table 4).

**Table 4:** Comparison of participants’ laboratory variables, reactive hyperemia index and blood pressure levels according to the diagnosis of hyperuricemia

	Control group (n = 130)	Hyperuricemia group (n = 19)	p	p*
**Metabolic Variables**				
Glucose (mg/dL)	86.00 (79.50 – 93.00)	87.00 (81.00 – 101.00)	0.51	0.78
Insulin (µU/mL)	12.28 (8.84 – 16.95)	12.70 (9.80 – 18.96)	0.41	0.59
HOMA-IR	2.61 (1.85 – 3.64)	2.68 (2.15 – 3.70)	0.41	0.39
Total cholesterol (mg/dL)	191.35 ± 40.55	194.47 ± 30.97	0.75	0.63
HDL-cholesterol (mg/dL)	52.00 (43.00 - 59.00)	43.00 (39.00 – 51.00)	0.01	0.17
LDL-cholesterol (mg/dL)	112.00 (89.00 – 140.00)	122.00 (96.00 – 145.00)	0.41	0.47
Triglycerides (mg/dL)	98.50 (68.00 – 142.00)	132.00 (108.00 – 142.00)	0.15	0.45
**Inflammatory Profile**				
Hs-CRP (mg/L)	0.37 (0.19 – 0.65)	0.45 (0.33 – 0.63)	0.24	0.70
Adiponectin (mg/mL)	5.65 (4.27 – 8.37)	4.02 (3.26 – 5.53)	0.04	0.11
**Oxidative Stress**				
Malondialdehyde (ng/mL)	3.53 (3.10 – 4.07)	4.67 (4.03 – 5.30)	0.0004	< 0.0001
**Endothelial Function**				
Reactive hyperemia index	2.05 ± 0.46	1.68 ± 0.30	0.005	0.03
**Blood Pressure**				
Systolic BP (mmHg)	119.67 (104.00 – 127.00)	121.30 (109.30 – 132.30)	0.23	0.46
Diastolic BP (mmHg)	76.76 ± 11.57	78.81 ± 8.63	0.46	0.28
Heart Rate (bpm)	74.00 (69.00 – 80.17)	69.00 (64.33 – 76.33)	0.10	0.16

Values as mean ± standard deviation for normal distribution or as median (interquartile interval) for not normal distribution. HOMA-IR, homeostasis model assessment of insulin resistance; HDL: high density lipoprotein; LDL: low density lipoprotein; Hs-CRP: high-sensitivity C-reactive protein; BP: blood pressure. p: Control group vs.Hyperuricemia group.

* p: Control group vs.Hyperuricemia group, after adjustment for age, sex and body mass index

The evaluation of endothelial function revealed significantly lower values of RHI in the hyperuricemia group than in control group even after adjustments for confounders. Mean values of systolic and diastolic blood pressure were similar in both study groups (Table 4).

Considering data from all participants (n = 149), correlation analyses of SUA with laboratory variables, blood pressure and endothelial function revealed some significant associations (Table 5). SUA was directly associated with BMI, WC, glucose, total cholesterol, LDL-cholesterol, TG, MDA, systolic blood pressure and diastolic blood pressure. It was inversely correlated with HDL-cholesterol, adiponectin and RHI. After adjustment for age and sex the association of uric acid with BMI and WC remained significant. The positive associations of SUA with triglycerides and MDA, and negative associations with HDL-cholesterol, adiponectin, and RHI also remained significant after adjustment for age, sex and BMI (Table 5).

**Table 5:** Correlations between serum levels of uric acid and biochemical variables, reactive hyperemia index and blood pressure (n = 149)

	Correlation		Partial correlation*	
	r	p	r	p
**Anthropometric Parameters**				
Body mass index (kg/m^2^)	0.39	< 0.0001	0.30	0.0003
Waist circumference (cm)	0.43	< 0.0001	0.26	0.001
**Metabolic Variables**				
Glucose (mg/dL)	0.21	0.01	0.25	0.08
Insulin (µU/mL)	0.01	0.94	0.03	0.82
HOMA-IR	0.07	0.62	0.07	0.64
Total cholesterol (mg/dL)	0.22	0.01	0.14	0.10
HDL-cholesterol (mg/dL)	-0.42	< 0.0001	-0.28	0.0007
LDL-cholesterol (mg/dL)	0.29	0.0003	0.19	0.02
Triglycerides (mg/dL)	0.35	< 0.0001	0.21	0.01
**Inflammatory Profile**				
Hs-CRP (mg/L)	0.11	0.23	0.16	0.10
Adiponectin (mg/mL)	-0.40	0.0005	-0.25	0.03
**Oxidative Stress**				
Malondialdehyde(ng/mL)	0.28	0.04	0.31	0.03
**Endothelial Function**				
Reactive hyperemia index	-0.27	0.01	-0.25	0.02
**Blood Pressure**				
Systolic BP (mmHg)	0.32	0.0001	0.16	0.06
Diastolic BP (mmHg)	0.24	0.003	0.16	0.11

HOMA-IR, homeostasis model assessment of insulin resistance; HDL: high density lipoprotein; LDL: low density lipoprotein; Hs-CRP: high-sensitivity C-reactive protein; BP: blood pressure.

* After adjustment for age and sex (for the partial correlations with body mass index and waist circumference) or after adjustment for age, sex and body mass index (for the other variables).

## Discussion

In the present study carried out in healthy young and middle-aged adults, subjects with hyperuricemia, as compared to those without this condition, presented higher BMI, higher oxidative stress status, and worse endothelial function even after adjustments for potential confounders. In correlation analysis, after controlling for confounders, SUA levels were positively associated with BMI, WC, MDA, TG and LDL-cholesterol; and negatively correlated with HDL-cholesterol, adiponectin and RHI.

Previous cross-sectional studies have also observed a direct association between SUA and parameters of total and/or central body adiposity in individuals presenting different characteristics, such as obese postmenopausal women,^16^ patients with type 2 diabetes^17,18^ and individuals aged 18-70 years without type 1 or 2 diabetes.^3^ Accordingly, epidemiological longitudinal studies carried out in the general population, reported an association of higher levels of SUA and an increased risk of overweight/obesity.^19^

The mechanisms responsible for the relationship between elevated SUA and higher body adiposity are not completely understood. One possible explanation rests on the intake of fructose. The excessive consumption of fructose (via added sucrose or high-fructose corn syrup) stands as one of the dietary causes of hyperuricemia.^20^ There is evidence that fructose causes intracellular ATP depletion, nucleotide turnover, and generation of uric acid. The fructose-induced uric acid generation causes mitochondrial oxidative stress which can in turn, favor fat accumulation.^21,22^ Experimental studies also suggest that fructose intake may facilitate the development of overweight/obesity through other mechanisms, such as alteration in satiety and increase in food intake.^20,22^ Conversely, there are studies indicating that adipose tissue possesses abundant xanthine oxidoreductase activity (similar to liver) and is capable of generating and secreting uric acid: a property which is enhanced in obesity.^23^

A direct association between SUA and oxidative stress as reflected by serum levels of MDA was observed in the present study. This finding is in agreement with the hypothesis suggested by some authors that the relationship of SUA with vascular and metabolic derangements is, at least, partially mediated by alterations in oxidative stress.^21,24^ It is worth mentioning that the association of uric acid with oxidative stress is complex and may be paradoxical.^25^ Uric acid has the ability to induce intracellular and mitochondrial oxidative stress but is a major antioxidant in human plasma^25^ where it can account for roughly two-thirds of its total antioxidant capacity, through chelation of metals and oxygen radical scavenging.^20^ However, there is evidence that under ischemic conditions and when SUA is above normal levels it becomes a prooxidant.^24-26^ Xanthine oxidase, which is one of the two xanthine-oxireductase interconvertible isoforms, uses molecular oxygen as an electron acceptor, generating superoxide anion and other reactive oxygen species as byproducts, thereby raising oxidative stress which may ultimately contribute to CVD.^24,27^

Some studies, similarly to the present investigation, observed that SUA levels were related positively with TG^3,16,28^ and negatively with HDL-cholesterol.^3,18,28^ The mechanisms that underlie the relationship between SUA and TG are not yet known,^29^ but there are some possible explanations. According to one of them, uric acid can induce lipogenesis in the liver and can block fatty acid oxidation.^30,31^ Other investigators suggest that hepatic synthesis of fatty acids is associated with “de novo” synthesis of purine, with subsequent acceleration in uric acid production.^32^

In the present study hyperuricemia was associated with lower levels of serum adiponectin. Among the few studies that evaluated this association, one conducted by Park et al.^33^^)^ enrolled 841 postmenopausal women aged 50 years or older and found an inverse relationship, which was not reproduced in a cross-sectional analysis of Tromsø Study.^34^ Although serum levels of CRP-hs were not significantly associated with SUA, they were higher in individuals presenting hyperuricemia. A positive association between SUA and CRP was observed in some studies carried out in octagenarians,^35^ in postmenopausal women,^10^ in type 2 diabetics,^36^ in older persons^37^ and in obese prepubertal children.^38^ The impaired endothelial function observed in subjects with higher SUA levels in the present study was also found in previous studies.^4-6,9,11^ However, as previously mentioned, most of them enrolled older and sick individuals, in contrast to the present study, where healthy young and middle aged subjects were recruited.

According to Johnson et al.^39^ uric acid may be taken up by adipocytes, where it induces oxidative stress, generates inflammatory mediators and inhibits the synthesis of adiponectin.^39^ The potential increase in oxidative stress induced by SUA may also favor an inflammatory response and endothelial dysfunction through the reduction of nitric oxide bioavailability.^29^ There is evidence that SUA can also decrease nitric oxide production via others mechanisms.^38^

The strength of this study relies on the careful selection of participants, excluding individuals with characteristics that might influence SUA levels, as well as the metabolic and vascular markers evaluated here. For example, exclusions encompassed postmenopausal women and elderly, patients taking any type of medications (including diuretics), and those with hypertension, diabetes or chronic renal disease.^40^ It is not clear whether increased SUA is a causative agent or is simply a marker of CVD. The present study provides the information that even in healthy young and middle aged adults SUA is directly associated with oxidative stress and with metabolic and vascular alterations that may increase the risk of CVD. The limitation of this study is the cross-sectional design, implying that causality is not likely to be determined.

## Conclusions

The results obtained in this study suggest that in healthy young and middle-aged adults, higher serum levels of uric acid are associated with excessive body adiposity, worse lipid profile, oxidative stress, inflammation and impaired endothelial function.
